# Directional ionic transport across the oxide interface enables low-temperature epitaxy of rutile TiO_2_

**DOI:** 10.1038/s41467-020-15142-x

**Published:** 2020-03-16

**Authors:** Yunkyu Park, Hyeji Sim, Minguk Jo, Gi-Yeop Kim, Daseob Yoon, Hyeon Han, Younghak Kim, Kyung Song, Donghwa Lee, Si-Young Choi, Junwoo Son

**Affiliations:** 1 Department of Materials Science and Engineering (MSE), Pohang University of Science and Technology (POSTECH), Pohang, 37673 Republic of Korea; 2 Max Planck Institute of Microstructure Physics, Weinberg 2, Halle (Saale), 06120 Germany; 3 Pohang Accelerator Laboratory, Pohang, 37673 Republic of Korea; 4 Materials Modeling and Characterization Department, Korea Institute of Materials Science (KIMS), Changwon, Republic of Korea

**Keywords:** Electronic properties and materials, Surfaces, interfaces and thin films

## Abstract

Heterogeneous interfaces exhibit the unique phenomena by the redistribution of charged species to equilibrate the chemical potentials. Despite recent studies on the electronic charge accumulation across chemically inert interfaces, the systematic research to investigate massive reconfiguration of charged ions has been limited in heterostructures with chemically reacting interfaces so far. Here, we demonstrate that a chemical potential mismatch controls oxygen ionic transport across TiO_2_/VO_2_ interfaces, and that this directional transport unprecedentedly stabilizes high-quality rutile TiO_2_ epitaxial films at the lowest temperature (≤ 150 °C) ever reported, at which rutile phase is difficult to be crystallized. Comprehensive characterizations reveal that this unconventional low-temperature epitaxy of rutile TiO_2_ phase is achieved by lowering the activation barrier by increasing the “effective” oxygen pressure through a facile ionic pathway from VO_2-δ_ sacrificial templates. This discovery shows a robust control of defect-induced properties at oxide interfaces by the mismatch of thermodynamic driving force, and also suggests a strategy to overcome a kinetic barrier to phase stabilization at exceptionally low temperature.

## Abstract (web summary)

The research to utilize chemical potential mismatch for materials synthesis has been limited across the oxide interface. Here, the authors show that directional ionic transport from the VO_2_ layers stabilizes the rutile TiO_2_ phase at extremely low temperatures, at which epitaxy is difficult, by effectively lowering the activation barrier for crystallization.

## Introduction

**Fig. 1:**
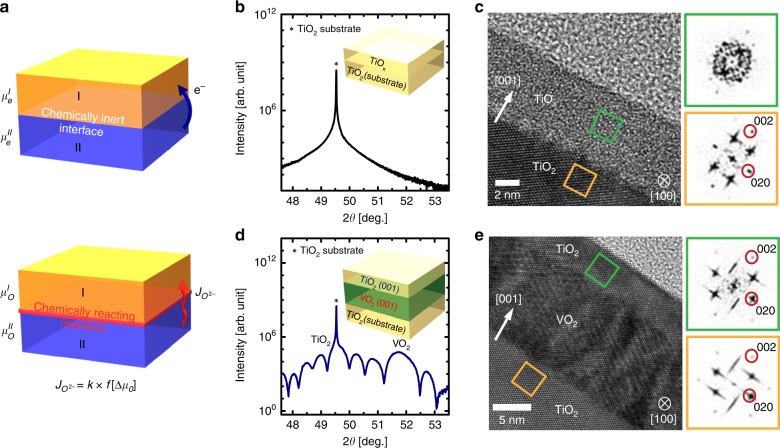
Low-temperature epitaxy of rutile TiO_2_ on VO_2_ sacrificial templates. **a** Schematics of possible directional charge (top) and ionic (bottom) transport due to chemical potential mismatch (Δ*μ*) across the interface with loss of translational symmetry. Symmetrical x-ray scan of **b** TiO_X_/TiO_2_ homostructure and **d** TiO_2_/VO_2_/TiO_2_ heterostructure containing TiO_2_ films grown at 150 °C. Contrary to the absence of peak around TiO_2_ substrate peak in homostructure (**b**), (002) Bragg reflections and Kiessig fringes around the peak from TiO_2_ substrate in heterostructure in **d** indicate that the TiO_2_ films are epitaxially grown at 150 °C on VO_2_ templates with sharp interface. HRTEM images and FFT patterns of **c** TiO_X_ /TiO_2_ homostructure (scale bar = 2 nm) and **e**. TiO_2_/VO_2_/TiO_2_ heterostructure (scale bar = 5 nm) projected with [100] zone axis. Unlike the amorphous nature of TiO_X_ films in homostructures (green square in **c**), obvious diffraction spots were observed in the FFT pattern of the TiO_2_ films on VO_2_/TiO_2_ (green square in **e**), and were same as those observed from the TiO_2_ substrates (yellow square in **e**); this similarity indicates an identical epitaxial relationship of TiO_2_ epitaxial films with TiO_2_ substrates in TiO_2_/VO_2_/TiO_2_ heterostructure.

Interfaces formed by two dissimilar materials can break the translational symmetry and thereby provide an opportunity to develop functionality that is unachievable in bulk materials^1–5^. When two materials (*I*, *II*) that have different work functions (i.e., $\mu _e^I \, < \, \mu _e^{II}$) are brought together at a semiconductor heterojunction, charge carriers near the interface diffuse across the junction (Fig. 1a);^1,2^ as a consequence, a conducting channel with high carrier density and high electron mobilities (e.g., two-dimensional electron gas) could be created at the interfaces between normally-insulating materials^2,3^. If an external bias is applied to adjust this built-in potential, the amount of transferred charge flow can be controlled by changing the electrochemical potential across chemically-inert interfaces^2,6^, which is the basic principle of heterojunction field effect transistors (HFETs)^7^.

As an analogy to the reversible control of electric charge transfer at an interface with discontinuity, (electro-)chemical potential mismatch for oxygen (Δ*μ*_*O*_) between two materials ($\mu _O^I \, < \, \mu _O^{II}$) may give rise to charged ionic transfer to bring the equilibrium of the system with heterogeneous junction at the interface (Fig. 1a)^5,8–12^. In particular, charged ionic defects migrate by ionic diffusion (e.g., diffusion of oxygen ions through vacancies) to mitigate Δ*μ*_*O*_ at the interface;^9–14^ charged ions, in principle, are transferred to adjacent materials and reconfigured by the redox reaction across the chemically-reacting interfaces in oxide heterostructure until the chemical potentials of the layers match ($\mu _O^I = \mu _O^{II}$ in Fig. 1a)^5^.

For example, the vanadium dioxide (VO_2_), the archetypal correlated oxide with metal-insulator (MI) transition near room temperature, is interfaced with ionic liquid (IL), and then (electro-)chemical potential can be adjusted by applying an external electric field across the VO_2_/IL interfaces^9,14–16^. In this case, instead of electric charge transfer, charged oxygen ions out-diffused to the IL to equilibrate the (electro-)chemical potential between VO_2_ and IL; the formation of oxygen vacancies (*V*_O_) by oxygen ion migration are responsible for the reversible insulator-to-metal transition and giant lattice expansion in VO_2_ films under the positive bias^15^. Furthermore, *V*_*O*_ concentrations that develop in LaNiO_3_, LaTiO_3_, and In_2_O_3_ can be modulated by the directional oxygen flow to the adjacent layers^10–12^.

At interfaces where ionic flux ($J_{O^{2 - }}$ in Fig. 1a) is directional, the dynamics of charged ions may be important by assembling other metal oxides that have different *μ*_*O*_^10,11,14^. The redistribution of charged vacancies can screen the electric fields that Δ*μ*_*O*_ causes. Therefore, the massive redistribution of charged ions can be accelerated by extremely increasing the thermodynamic Δ*μ*_*O*_ across the interfaces; by supplying unidirectional charged ionic flux, this redistribution may offer a spontaneous route that can facilitate synthesis of crystalline materials^12^, and may enable robust control of defect-induced properties at oxide interfaces^9,13,14^.

Here, we demonstrate the formation of high-quality rutile TiO_2_ epitaxial films at exceptionally low temperature, which is driven by directional transport of oxygen ions across the TiO_2_/VO_2_ heterointerfaces. Contrary to the amorphous nature of TiO_2_ films directly grown on TiO_2_ substrates at 150 °C, single-crystal rutile TiO_2_ layer is synthesized by forming the heterointerface with VO_2_ template at the lowest growth temperature *T*_G_ (<150 °C) ever reported, at which rutile TiO_2_ is difficult to be crystallized. By experimental characterization using atomic-resolution electron microscopy and synchrotron x-ray spectroscopy combined with theoretical calculation, we demonstrate that a facile ionic diffusion of oxygen ions from the oxygen reservoir VO_2_ along the [001] channel decreases Δ*μ*_*O*_, and thereby enables this unprecedented epitaxy of rutile TiO_2_ at low temperature by lowering the activation barrier for formation of stable nuclei. Interestingly, this directional ionic transport improves the registry in the lattice of TiO_2_ films at the expense of the structural and electronic modulation in an oxygen-deficient VO_2-δ_ sacrificial layer. As a result of the mismatch of thermodynamic driving force combined with kinetically-facile migration of oxygen ions across the TiO_2_/VO_2_ interfaces, the massive redistribution of oxygen ions enables low-temperature epitaxy of high-temperature-stabilized phase by increasing an “internal” oxygen supply across the chemically-reacting oxide interface.

## Results

### Low temperature epitaxial growth of rutile TiO_2_ films on VO_2_ template

Prior to TiO_2_ growth, the substrates with 12-nm-thick VO_2_ template were prepared on (001)-oriented TiO_2_ substrates by pulsed laser deposition (Supplementary Fig. 1). X-ray diffraction (XRD) results (Supplementary Fig. 2a) showed sharp VO_2_ (002)_R_ peaks (in rutile notation) at ~ 2*θ* = 65.9° (*c* = 0.2839 nm) without other peaks related to vanadium oxides that had valence states other than +4. A steep MI transition (Δ*R*_*S*_ ~ 10^3.3^) occurred on the VO_2_ films at *T*_*MI*_ ~ 298 K (Supplementary Fig. 2b); this result indicates the formation of coherently tensile-strained VO_2_ films with high crystal quality and negligible *V*_*O*_^9,15,17,18^.

Then, the 6 nm-thick TiO_2_ films were grown at low *T*_G_ ~ 150 °C with the same oxygen pressure ($pO_2$ ~ 12 mTorr) on two substrates: 1) (001) TiO_2_ single crystals without the VO_2_ template layer (denoted as TiO_2_/TiO_2_ hereafter) and 2) (001) TiO_2_ single crystals with the VO_2_ template (denoted as TiO_2_/VO_2_/TiO_2_). Symmetric 2θ-ω scans using synchrotron X-ray scattering on TiO_2_/TiO_2_ grown at *T*_G_ = 150 °C (Fig. 1b) detected no Bragg reflections except for substrate (2θ = 49.54°); this absence indicates no formation of crystalline TiO_2_ films^16^; the formation of amorphous TiO_2_ films on TiO_2_ substrates is attributed to the thermodynamic or kinetic instability of the rutile TiO_2_ phase, which requires sufficient thermal energy for phase formation^19–23^. To exclude the possible coincidence of diffraction peak from TiO_2_ films and substrates in homostructures in Fig. 1b, TiO_2_ films were also grown on (100) Al_2_O_3_ single crystal substrates (Supplementary Fig. 3). The symmetric 2θ-ω scan in wide range of angle also detected the only peak related to the (100) Al_2_O_3_ substrate (2θ = 68.22°) due to the formation of amorphous films at *T*_G_ = 150 °C.

In contrast, rutile TiO_2_ epitaxial films were strikingly stabilized by introducing the VO_2_ layers on TiO_2_ substrate at the same condition with *T*_G_ = 150 °C. Symmetric 2θ-ω scans of TiO_2_/VO_2_/TiO_2_ heterostructures (Fig. 1d) showed two Bragg reflections, one from the rutile TiO_2_ (2θ = 49.46°) substrates, and one from VO_2_ (2θ = 51.8°) films. More importantly, the TiO_2_ substrate peak was resolved to a slightly broad peak from TiO_2_ epitaxial films^16^, which did not appear in the scans of TiO_2_/TiO_2_ homostructure. Each film peak exhibited a Kiessig fringe; fitting of the peaks showed that their periodicity differed due to different film thickness (Supplementary Fig. 4); these clear oscillations from peaks represent sharp interface of TiO_2_/VO_2_/TiO_2_ all-epitaxial heterostructures.

The low-temperature epitaxy of rutile TiO_2_ films on VO_2_-templated substrates was locally visualized by comparing cross-section high-resolution transmission electron microscope (HRTEM) images of both TiO_*x*_ (*x* < 2)/TiO_2_ and TiO_2_/VO_2_/TiO_2_ (Fig. 1c, e). Amorphous nature of TiO_x_ films on TiO_2_ was confirmed by the diffused halo feature in fast Fourier transform (FFT) pattern from the films (green square in the right column of Fig. 1c). In contrast, sharp diffraction spots were observed in FFT pattern of the TiO_2_ films on VO_2_/TiO_2_ (green square in the right column of Fig. 1e), and is similar with that from the TiO_2_ substrates (yellow square in the right column of Fig. 1e); this observation demonstrates that epitaxial rutile TiO_2_ films can be crystallized at 150 °C simply by introducing VO_2_ templates on TiO_2_ substrate. Considering the dramatic difference of crystallinity in those TiO_2_ films grown at the same growth condition, the epitaxial TiO_2_ with excellent crystallinity at 150 °C is unusual, because it formed even though thermal energy was insufficient at 150 °C to overcome the activation energy that is required to drive formation of thermodynamically stable crystalline nuclei^20,23^.

**Fig. 2:**
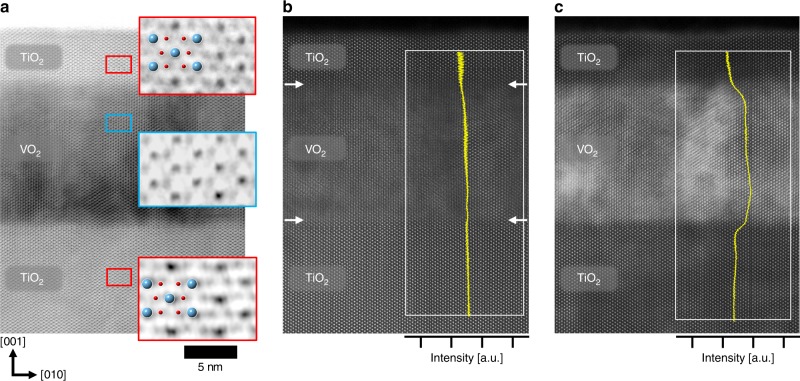
Atomic-resolution analysis of TiO_2_/VO_2_/TiO_2_ heterostructure. **a** ABF-STEM, **b** HAADF-STEM and **c** LAADF-STEM images of rutile TiO_2_ epitaxial film grown on VO_2_ sacrificial template at *T*_G_ = 150 °C with [100] zone axis (scale bar = 5 nm). The sensitivity of ABF technique to light-weight atoms enables observation of oxygen atoms along the [100] zone axis. Note that the regular pattern from TiO_2_ epitaxial films (top red square in **a**) was identical to that from TiO_2_ substrates (bottom red square in **a**), indicating an identical atomic arrangement of films with single crystals by epitaxial growth without oxygen defects. On the contrary, the oxygen-deficient region was observed in the sacrificed VO_2_ template near the TiO_2_ film (blue square in **a**). While almost-uniform HAADF contrast was observed across the heterostructures due to similar cation atomic weight across the heterostructures (**b**), a noticeable strain-field-induced LAADF contrast was observed in VO_2_ templates (**c**) sandwiched between TiO_2_ films and substrates. Yellow lines in **b** and **c** are the contrast-intensity profiles of the HAADF and LAADF images from the white rectangular areas.

To determine how this unprecedented rutile TiO_2_ phase developed low-temperature epitaxy, annular bright field (ABF) scanning transmission electron microscopy (STEM) data were analyzed with TiO_2_/VO_2_/TiO_2_ heterostructure. The sensitivity of ABF to light-weight atoms permitted visualization of oxygen atomic columns in ABF STEM (Fig. 2a)^17^. Magnified ABF-STEM images (red rectangles) show the typical rutile TiO_2_ structure in both film and substrate; this result indicates that TiO_2_ films had been fully crystallized by coherent epitaxial growth on VO_2_ templates. However, the contrasts of oxygen atomic columns in VO_2_ are weak and diffuse in the enlarged images of VO_2_ (blue rectangle); this result implies that oxygen contents are deficient in the VO_2_ template after low-temperature epitaxy of stoichiometric rutile TiO_2_ films. Therefore, TiO_2_ films with perfect registry of atoms were epitaxially grown on top of defective VO_2_ templates; this result is contrary to the general principle that high-quality epitaxial growth is achieved by using low-defect substrates, and suggests that the chemical reaction at the interface is likely to facilitate the low-temperature epitaxy of high-quality TiO_2_ films by sacrificing the initially good quality of VO_2_ templates.

The formation of defective features in VO_2_ template could be confirmed by comparing high-angle annular dark field (HAADF) and low-angle annular dark field (LAADF) signals of TiO_2_/VO_2_/TiO_2_ heterostructures from STEM. Contrary to almost identical HAADF contrast across the heterostructures due to similar cation atomic weight across the heterostructures^16^ (Fig. 2b), the LAADF contrast was noticeably mottled in VO_2_ templates (Fig. 2c) that were sandwiched between TiO_2_ films and substrates. The HAADF and LAADF images had distinct intensity profiles along the film growth direction of [001] (insets in Fig. 2b, c) The differences occur because the LAADF signal is more sensitive than the HAADF signal to the frustrated atomic channeling due to oxygen vacancies (*V*_*O*_)^4,24^, so the contrast is blurred and brighter in the LAADF signal of VO_2_ templates. Moreover, electron energy loss spectroscopy (EELS) experiments reveal that the *t*_2g_ peak of O-*K* edge was strongly suppressed in the entire VO_2_ template (Supplementary Fig. 5b); this result directly visualize the formation of oxygen vacancies^25^. Therefore, the combined results from LAADF contrast and EELS data in VO_2_ templates confirms the significant loss of oxygen atoms from VO_2_ during the low-temperature epitaxial growth of TiO_2_ films in the heterostructures.

### Suppression of metal-insulator transition in VO_2_ templates by directional oxygen transport

**Fig. 3:**
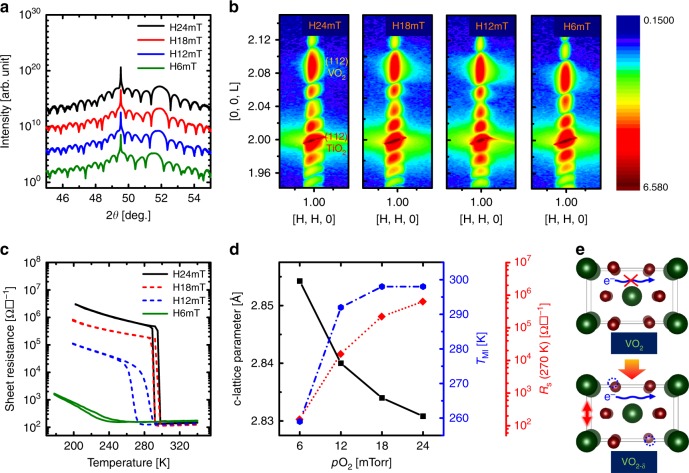
Structural/electronic modulation by directional ionic transport. **a** Symmetrical X-ray scan and **b** reciprocal space mapping around (112) reflection of TiO_2_ grown under $pO_2$ = 6 mTorr (denoted as H6mT) ~ 24 mTorr (denoted as H24mT) on VO_2_-templated TiO_2_ substrates. These results confirm that entire layers in all heterostructures are coherently strained by TiO_2_ substrates, but only VO_2_ peaks shifted to lower scattering angles as $pO_2$ decreased during TiO_2_ growth. **c** Temperature-dependent sheet resistance (*R*_S_) in all TiO_2_/VO_2_/TiO_2_ heterostructures with TiO_2_ epitaxial films grown at 6 ≤ $pO_2$ ≤ 24 mTorr on VO_2_-templated TiO_2_ substrates. **d** Lattice parameter (*c*) from **a** and temperature (*T*_MI_) of metal-insulator transition and *R*_S_ at 270 K from **c** as a function of $pO_2$ during TiO_2_ growth. **e** The formation of oxygen vacancies in VO_2_ by ionic transfer across the TiO_2_/VO_2_ interface expanded the lattice to compensate for the larger cation radius of V^3+^ (3*d*^2^) than V^4+^ (3*d*^1^), and also led to the oxygen-vacancy-induced metallization.

Interestingly, the degree of oxygen deficiency of VO_2_ templates was sensitively modulated by adjusting $pO_2$ (6 mTorr ~ 24 mTorr) during TiO_2_ growth at *T*_G_ ~ 150 °C on VO_2_-templated TiO_2_ substrates. As observed in symmetric 2θ-ω synchrotron XRD scans in all heterostructures, Bragg peaks and Kiessig fringes from TiO_2_ films were resolved from those from TiO_2_ substrates and VO_2_ templates with different period of oscillations in fringes (Fig. 3a), which again confirms the importance of VO_2_ templates for low-temperature epitaxy of TiO_2_ layers. However, unlike the almost identical peak of TiO_2_ films and substrates, the (002) reflection of 14-nm-thick VO_2_ template films decreased from 2θ = 51.9° (black) to 2θ = 51.5° (green) from symmetric 2θ-ω scans as $pO_2$ was reduced from 24 mTorr to 6 mTorr during the TiO_2_ growth (Fig. 3a); this peak shift corresponds to ~ 0.8 % expansion of the out-of-plane lattice parameters in VO_2_ templates.

For more detailed structural modulation of TiO_2_/VO_2_/TiO_2_ heterostructures, reciprocal space mapping (RSM) around the (112) reflection of (001) TiO_2_ substrate was performed to obtain the information on both in-plane and out-of-plane lattice parameters by adjusting $pO_2$ during TiO_2_ growth (Fig. 3b). The RSM data of all heterostructures clearly show sharp and intense (112) Bragg reflections and Kiessig fringes from TiO_2_ substrate and film, and from the VO_2_ films. The substrate and film peaks showed identical H (i.e., in-plane reciprocal space unit)^26,27^, which implicates that entire layers in all heterostructures are coherently strained by TiO_2_ substrates along the in-plane direction. Geometric phase analysis (GPA) strain quantification using obtained STEM image confirms coherent interfaces through the heterostructures (Supplementary Fig. 6). However, only the VO_2_ peak shifted to lower scattering angle (i.e., characteristic of expansion of out-of-plane lattice parameters) as the $pO_2$ during TiO_2_ growth was decreased; these trends are consistent with vanadium valence state switching (V^4+^ to V^3+^) by the formation of *V*_*O*_ in VO_2_ templates^15,16,28^.

Temperature-dependent sheet resistance *R*_S_ (T) of TiO_2_/VO_2_/TiO_2_ heterostructures (by van der Paw methods) was measured to quantify how the accelerated oxygen deficiency in VO_2_ templates affected electrical transport of the heterostructures (Fig. 3c). The heterostructure that had been formed using $pO_2$~ 24 mTorr during TiO_2_ growth (denoted as H24mT hereafter) exhibited slightly suppressed MI transition in terms of *R*_*S*_(T) (i.e., ~ 3.2 orders of magnitude at *T*_*MI*_ ~ 298 K) compared with as-grown VO_2_ films without TiO_2_ layers on top. On the contrary, *R*_S_(T) of the heterostructure with $pO_2$ of 6 mTorr (denoted as H6mT) substantially dropped by just less than an order of magnitude; This result indicates that MI transition of VO_2_ templates was progressively suppressed and *T*_*MI*_ was monotonically decreased from 298 K (H24mT) to 260 K (H6mT) by the gradual increase of *V*_*O*_ in VO_2_ as the TiO_2_ films were grown at progressively lower $pO_2$ (Fig. 3d)^4,9^. As a result, the formation of *V*_*O*_ by oxygen ionic transfer across the interface not only expanded the lattice to compensate for the larger cation radius of V^3+^ (3*d*^2^) than V^4+^ (3*d*^1^)^28^, but also induced the metallic state at TiO_2_/VO_2_ interfaces even at 270 K (Fig. 3e). Structural and electrical modulation driven by *V*_*O*_ in VO_2_ cannot be generated by simple post-annealing without TiO_2_ layer growth on top; directional oxygen ionic transport indeed occurs across the TiO_2_/VO_2_ interfaces by forming *V*_*O*_ in VO_2_ layer, as long as TiO_2_ layers are grown on VO_2_ templates (Supplementary Fig. 14).

**Fig. 4:**
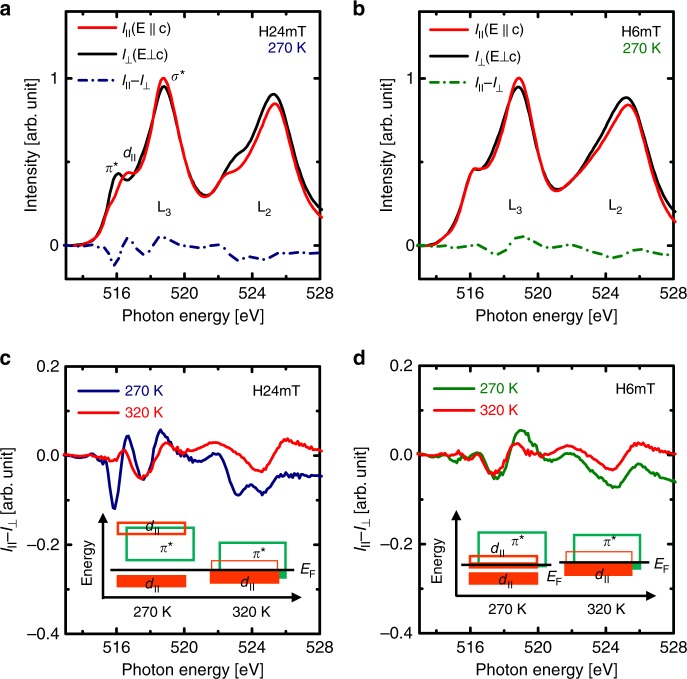
Oxygen-vacancy-induced isotropic orbital occupancy in VO_2_ templates. Polarization-dependent x-ray absorption spectroscopy (XAS) at the V *L*_*2,3*_-edges at 270 K for two heterostructures composed of 2.5-nm-thick TiO_2_ films grown on VO_2_ templates under different $pO_2$ (**a** H24mT, **b** H6mT). Unlike large difference of XAS signal in H24mT at 270 K due to the orbital polarization with V-V dimerization in the monoclinic VO_2_, almost no effect on the XAS signal in H6mT was observed at 270 K; this result indicates isotropic orbital filling in H6mT sample even at 270 K. The XLD (*I*_||_ – *I*_⊥_) are also shown at both 270 K and 320 K for **c** H24mT and **d** H6mT. Oxygen vacancies driven by directional ionic transport in H6mT tend to increase the crystal symmetry to close to rutile structure by weakening of V-V dimerization, so selective filling of *d*_||_ (inset of **c**.) changes to isotropic orbital occupancy of *d*_||_ and π* (inset of **d**) in VO_2_ templates; the oxygen-vacancy-driven isotropic occupancy leads to metallization at 270 K.

To elucidate the origin of metallicity in a VO_2_ template interfaced with a TiO_2_ layer, we performed polarization-dependent x-ray absorption spectroscopy (XAS) at the V *L*_*2,3*_-edges for two heterostructures that contained 2.5-nm-thick TiO_2_ films grown on VO_2_ templates under $pO_2$ = 24 mTorr (H24mT, Fig. 4a) and 6 mTorr (H6mT, Fig. 4b). The XAS signals at the V *L*_*2,3*_-edges represent a dipole-allowed transition from the V 2*p*_1/2_ and 2*p*_3/2_ core level to the V 3*d* valence electronic states (i.e., 2*p*^6^3*d*^1^ → 2*p*^5^3*d*^2^)^29–31^ only from the VO_2_ templates buried under layers of rutile TiO_2_ owing to its element-specific character. Linearly-polarized X-rays with the polarization vector parallel (**E***||c*) and perpendicular (**E**⊥c) to the out-of-plane orientation (*c* axis), respectively, detect the vacant *d*_||_ and π* electron states^31^, so *V*_*O*_ formation also significantly affects the dichroic signal (Fig. 4a, b) related to selective orbital occupancy of *d*_||_ induced by V-V dimerization^30,31^.

In H24mT sample, XAS spectra collected at 320 K (*T* > *T*_*MI*_) were similar regardless of the polarization direction of the X-ray (Supplementary Fig. 7a); this result was expected because of the isotropic orbital filling in the metallic states of VO_2_ due to absence of V-V dimerization. At 270 K (*T* < *T*_*MI*_, Fig. 3c), X-ray linear dichroism (XLD, *I*_||_ – *I*_⊥_) was much larger than at *T* = 320 K (Fig. 4a, c); this increase is a signature of orbital polarization, which is expected to result from the strong V-V dimerization in the insulating states, due to the selective filling of *d*_||_ orbitals in VO_2_ films with negligible oxygen loss (inset, Fig. 4c)^30,31^. In contrast, XAS spectra of H6mT, which is the sample with the highest driving force for oxygen ion transport from VO_2_ sacrificial template, show no polarization-dependence of incident X-ray (Fig. 4d) at either *T* = 270 K (Fig. 4b) or 320 K (Supplementary Fig. 7b). This result indicates that selective filling of *d*_||_ orbitals did not occur below 270 K^30,31^ and explains the *V*_*O*_-induced metallic behavior at 270 K in H6mT: *V*_*O*_ tends to increase the crystal symmetry toward thermally-induced tetragonal rutile structure by weakening of V-V dimerization^15,32,33^ and leads to isotropic orbital occupancy of *d*_||_ and π*^33^, (inset of Fig. 4d). The absence of selective filling in *d*_||_ orbitals in H6mT samples provides strong evidence for *V*_*O*_ formation in the entire area of sacrificial VO_2_ templates by directional oxygen transport from VO_2_ to TiO_2_.

### Stoichiometric TiO_2_ epitaxy induced by directional oxygen transport

**Fig. 5:**
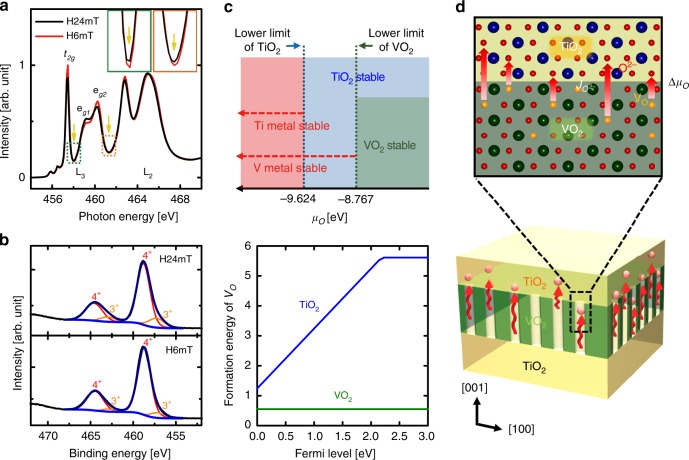
Facilitated rutile TiO_2_ epitaxy by Δ*μ*_*O*_ across the TiO_2_/VO_2_ interface. **a** Ti *L*-edge XAS spectra of TiO_2_ grown on VO_2_ templates under different $pO_2$ (H24mT, H6mT). The Ti *L*_*2,3*_-edge XAS signals from the H6mT were more intense and sharper than from H24mT. **b** XPS spectra of the Ti 2*p* core level of H24mT, H6mT. negligible Ti^3+^ contribution from the H6mT was observed compared to H24mT in TiO_2_/VO_2_ heterostructures, which reveals the suppression of *V*_*O*_ formation even at the surface of TiO_2_ as a result of increased oxygen transport across TiO_2_/VO_2_ interface under low $pO_2$. Both XAS and XPS results reveal increased perfection of rutile TiO_2_ films after growth at low $pO_2$. **c** First-principles density functional theory (DFT) calculations to determine values of the lower and upper limit of the chemical potential of *μ*_*O*_ for TiO_2_ and VO_2_ formation. TiO_2_ is the only stable compound at − 9.624 eV ≤ *μ*_*O*_ ≤ − 8.767 eV. Comparison of the formation energies of *V*_*O*_ in rutile VO_2_ and TiO_2_ as a function of Fermi level in the band gap of TiO_2_. **d** Increased thermodynamic driving force Δ*μ*_*O*_, assisted by high ionic kinetics *k*, across the interface increased the perfection of registry in the lattice of TiO_2_ films by increasing “effective” $pO_2$ and lowering the activation barrier for epitaxy with concurrent emergence of a metallic VO_2-δ_ sacrificial templates.

To explore the influence of directional ionic transport on the quality of TiO_2_ epitaxial layer grown at *T*_*G*_ ~ 150 °C, we evaluated the element-specific Ti *L*_*2,3*_-edge XAS signal from the TiO_2_ layer in TiO_2_/VO_2_ heterostructures. The Ti *L*_*3*_ edge peak between 457.9 eV ~ 461.3 eV (i.e., related to *e*_g_ orbitals) is split into two peaks due to the distortion of the TiO_6_ octahedra in rutile structure; the relative intensities of these *e*_g_ doublet (*e*_g_^1^ < *e*_g_^2^) verified a rutile TiO_2_ phase in both H24mT and H6mT films^34,35^ (Fig. 5a**)**, which is consistent with our results in HRXRD and STEM. The Ti *L*_*2,3*_-edge signals from H6mT were more intense and sharper than from H24mT^36,37^. Furthermore, the contribution of Ti^3+^ *L*-edge signals slightly increased the dips at 458 eV and 461 eV in the H24mT relative to those in H6mT (inset of Fig. 5a, yellow arrow);^36^ these results reveal that rutile TiO_2_ films toward the stoichiometry with low oxygen deficiency can be formed more easily by the TiO_2_ growth with low $pO_2$ than with high $pO_2$. Moreover, EELS data from the top TiO_2_ layers in H6mT show that the *t*_2g_ peaks of Ti-*L* edge from TiO_2_ layers is exactly same as those from stoichiometric bulk TiO_2_ substrates (reference) (Supplementary Fig. 5c); this result represents the formation of stoichiometric TiO_2_ layers at the expense of oxygen deficiency in VO_2_ layers.

In addition to bulk-sensitive XAS and EELS, X-ray photoelectron spectroscopy (XPS) of the Ti 2*p* core level shows better stoichiometry of rutile TiO_2_ film surface grown on VO_2_ templates under low $pO_2$ than under high $pO_2$ (Fig. 5b). Deconvolution of The Ti 2p core-level peaks with Ti^4+^ (2*p*_3/2_ ~ 458.8 eV, 2*p*_1/2_ ~ 464.6 eV) and Ti^3+^ (2*p*_3/2_ ~ 457.2 eV, 2*p*_1/2_ ~ 463.1 eV) valence states^38^ showed negligible Ti^3+^ contribution from the H6mT was observed compared to H24mT in TiO_2_/VO_2_ heterostructures, which reveals the suppression of *V*_*O*_ formation even at the surface of TiO_2_ resulting from enhanced oxygen transport across TiO_2_/VO_2_ interface under low $pO_2$; Both XAS and XPS results contradict the typical observation that stoichiometry could be improved in TiO_2_ under high $pO_2$ by removing *V*_*O*_^21,39^.

## Discussion

This study presents two interesting observations. (1) The increase in directional oxygen transport from VO_2_ to TiO_2_ with decrease in $pO_2$ during TiO_2_ growth, and (2) facile formation of rutile TiO_2_ epitaxial layer at extremely low temperature (≤150 °C). To identify the driving force for spontaneous oxygen loss from VO_2_ templates, firstly, density functional theory (DFT) calculations were performed to determine values of the lower and upper limit of the chemical potential of oxygen (*μ*_*O*_) for TiO_2_ and VO_2_ formation in general:

1 $$\frac{1}{2}\left( {E_{tot}\left[ {TiO_2(or\,VO_2)} \right] - \mu _{Ti}\left[ {Ti(or\,V)} \right]} \right) \le \mu _O\left[ {TiO_2(VO_2)} \right] \le \mu _O[O_2].$$

Our calculations predict that the lower limit of *μ*_*O*_ is −8.767 eV for VO_2_ and −9.624 eV for TiO_2_ (top of Fig. 5c), which indicates that TiO_2_ is the only stable compound at −9.624 eV ≤ *μ*_*O*_ ≤ −8.767 eV. In the specific *μ*_*O*_ region in which VO_2_ is thermodynamically unstable, oxygen atoms can preferentially migrate from it to stable TiO_2_.

Indeed, VO_2_ and TiO_2_ coexist at the interfaces in the heterostructures, so the following thermodynamic reactions occur during TiO_2_ growth:

2 $$TiO_{2 - \delta }\left( s \right) + VO_2\left( s \right) \leftrightarrow TiO_2\left( s \right) + VO_{2 - \delta }(s)$$

Thermodynamic calculations using this reaction yielded a Gibbs free energy ΔG = −18.69 kJ/mol at δ = 0.125, and −82.77 kJ/mol at δ = 0.5 at *T*_G_ = 150 °C^40–42^. Regardless of the degree of oxygen deficiency in grown TiO_2-δ_ layer, oxygen ions tend to transfer spontaneously to the TiO_2_ layer to equilibrate *μ*_*O*_ between the two layers by forming *V*_*O*_ in VO_2_ templates. A thermodynamic driving force between TiO_2-δ_ and VO_2_ still exists even if few monolayer of TiO_2_ prevents the direct interface between two dissimilar materials, so “remote” oxygen ionic transport from VO_2_ to TiO_2-δ_ is maintained through the few TiO_2_ monolayer as long as oxygen diffusion is kinetically allowed (Supplementary Figs. 9, 10, 15)^16,43^. To support our observation on the preferred formation of *V*_*O*_ in VO_2_ templates, we also compared the formation energies of *V*_*O*_ (and vanadium interstitials (*V*_*i*_)) in rutile VO_2_ and in TiO_2_ as a function of Fermi level in the band gap of rutile TiO_2_ (bottom of Fig. 5c). Our calculations predict that the formation energy of *V*_*O*_ is at least 0.69 eV lower in VO_2_ than in TiO_2_ and is ~ 2.0 eV lower that of *V*_*i*_ in VO_2_ (Supplementary Fig. 8).

It should be emphasized that the driving force for ionic flux accelerates with increase in the oxygen deficiency on the formed TiO_2-δ_ layers (i.e., decrease in $pO_2$ at which the film was grown) by maximizing chemical potential mismatch ($J_{O^{2 - }} \propto \Delta {\mathrm{G}}({\mathrm{or}}\,\Delta {\upmu})$)^10^. Due to this strengthened driving force for directional oxygen diffusion from VO_2_ to TiO_2_, more oxygen vacancies prefer to form in VO_2_ templates during TiO_2_ growth as $pO_2$ was decreased. Δ*μ*_*O*_ across TiO_2_/VO_2_ interfaces in the heterostructure drives oxygen flux ($J_{O^{2 - }}$) as a directional supply of oxygen ionic radical across the interface from VO_2_ sacrificial layers without the dissociation of oxygen gas molecules^10^. The transferred oxygen ions can “effectively” increase the oxygen partial pressure ($pO_2$) and *μ*_*O*_ at the TiO_2_ side; paradoxically, low $pO_2$ at the ambient is likely to increase “effective” $pO_2$ (= “external” $pO_2$ from O_2_ gas + “internal” $pO_2$ across the solid-solid interface) during TiO_2_ growth on VO_2_ templates. As a result, the increased “effective” $pO_2$ by the enhanced $J_{O^{2 - }}$ across the interface magnifies driving force for the formation of rutile TiO_2_ with stoichiometry in heterostructure (Supplementary Fig. 16); lack of oxidation in the deposited TiO_2-δ_ species during the growth is compensated by transferred oxygen ions from the VO_2_ templates below^10,44^.

For heterogenous nucleation on the substrate during the film growth, the activation energy (ΔG^*^) for the formation of crystalline nuclei could be significantly lowered by increasing the supply of oxygen ions (i.e., increasing the driving force for formation of rutile TiO_2_) based on the following expression^45^.

3 $$\Delta {\mathrm{G}}^ \ast = \frac{{16\pi \gamma _{fv}^3}}{{3\left( {\Delta {\mathrm{G}}_v - \Delta {\mathrm{G}}_S} \right)^2}}S(\theta )$$

where ΔG_*v*_, *γ*_*fv*_, ΔG_*S*_, and *S*(*θ*) are the chemical free energy change for the formation of solid rutile TiO_2_ nuclei, surface free energies, interfacial strain energy and a geometrical factor for heterogeneous nucleation, respectively. At very low *T*_G_ ~ 150 °C, the adatoms freeze in metastable form, so the time constant for crystallization $\big(\tau_{cryst}, \, {\mathrm{i.e.}}, \, \frac{1}{{\tau _{cryst}}} = {\mathrm{A}} \cdot {\mathrm{exp}}\big( { - \frac{{\Delta G^ \ast }}{{k_BT_g}}} \big)\big)$ becomes extremely long due to the high ΔG^*^ and insufficient thermal energy; the amorphized or metastable TiO_2_ films (with nonequilibrium structure) are likely to form due to the kinetic hindrance of crystal formation (e.g., insufficient movement of ablated TiO_2_ adatoms and/or limited reaction with O_2_ gas) as observed in our TiO_2_/TiO_2_ homostructure.

In our case, VO_2_ sacrificial layers from the bottom acts as epitaxial templates for rutile TiO_2_. They also sacrifice themselves by forming *V*_*O*_ in VO_2_ and thereby supply high concentration of oxygen ions to TiO_2-δ_ by magnifying the driving force for the oxygen transport at the VO_2_/TiO_2_ interfaces. Since *γ*_*fv*_ and ΔG_*S*_ are unlikely to be changed regardless of the existence of VO_2_ templates (Supplementary Fig. 6 and Fig. 15), greater ΔG_*v*_ induced by $J_{O^{2 - }}$ significantly reduce ΔG^*^ in heterostructure than in homostructure. As a result, the large reduction of *τ*_*cryst*_ by the decrease in ΔG^*^ enables unprecedented epitaxy of high-temperature-stabilized TiO_2_ rutile phase^19,23^ at extremely low *T*_G_ ~ 150 °C by changing the initial stoichiometry of two oxides with different *μ* across the interface.

In addition to thermodynamic viewpoint, the transport of charged oxygen ions is kinetically facilitated along the crystallographic [001] direction, which has open channels in anisotropic rutile VO_2_ and TiO_2_^15,16^. One-dimensional empty channels are aligned along the *c* axis in our TiO_2_/VO_2_ heterostructures and provide the advantage of removing significant amounts of oxygen ions due to a high oxygen diffusion coefficient. Thus, the growth direction of [001] TiO_2_ films should strongly accelerate the out-diffusion kinetics of oxygen transport (increased *k* in Fig. 1a) from the VO_2_ sacrificial templates as a result of mismatch in chemical potential (Δ*μ*_*O*_ in Fig. 1a); significant reduction in oxygen transport across the oxide interface along the [001] direction kinetically facilitates the decrease in ΔG^*^ for nucleation of rutile TiO_2_ phase even at low *T*_G_ to support epitaxial growth of rutile TiO_2_ films on VO_2_ templates.

On the other hand, the kinetics of oxygen transport will eventually limit our epitaxial growth based on “internal” oxygen transport across the TiO_2_/VO_2_ interface (Supplementary Figs. 11, 12, 13). Since the oxygens should be supplied from the TiO_2_/VO_2_ interfaces through the intervening TiO_2_ layers, the thickness of “epitaxial” TiO_2_ will be limited by oxygen diffusion through the intervening TiO_2_ layer. In fact, while the “epitaxial” thickness linearly increased with growth time in the TiO_2_ films grown at 300 °C, the “epitaxial” thickness appears to be saturated to be ~ 10 nm as a “critical” thickness at *t*_*film*_ > 10 nm at *T*_G_ ~ 150 °C due to scarce source of “internal” oxygen transport ($J_{O^{2 - }}$) even if the growth time increases (Supplementary Fig. 11**)**. Therefore, the existence of “critical” thickness provides the convincing evidence of our unprecedented low-temperature epitaxy driven by direction oxygen ionic transport.

In summary, unconventional low-temperature epitaxy of rutile TiO_2_ films was achieved by exploiting the directional transport of oxygen ions across TiO_2_/VO_2_ heterointerfaces. The thermodynamic driving force, assisted by facile ionic pathway along oxygen channel, across the interface enabled more perfect registry in the lattice of TiO_2_ films by lowering the activation barrier for stable nuclei, with concurrent emergence of a metallic TiO_2_/VO_2_ heterostructures. Contrary to typical experimental condition to obtain TiO_2_ with better stoichiometry, interestingly, *V*_*O*_ formation was diminished under low external $pO_2$, because the accelerated chemical potential mismatch (Δμ_*O*_) under low external $pO_2$ significantly increased “effective” $pO_2$ by the internal oxygen transport across the TiO_2_/VO_2_ interface. Therefore, the controlled ionic transport by Δ*μ*_*O*_ may offer an opportunity to design a new heterostructure with different degree of freedom at the interfaces as a result of tuning of ionic defects, and also to stabilize thermal-energy-requiring phases simply by interfacing with dissimilar materials with different thermodynamic and kinetic driving force of ionic defects.

## Methods

### Synthesis of epitaxial TiO_2_/VO_2_ heterostructures on TiO_2_ substrates

Epitaxial VO_2_ thin films (10–14 nm thick) were grown on (001) TiO_2_ single-crystal substrates, followed by the growth TiO_2_ films (2.5–60 nm thick) by pulsed laser deposition (PLD). The stoichiometric targets for the synthesis of heterostructures were prepared by sintering stoichiometric powders of V_2_O_5_ (99.99%, Sigma Aldrich) at 600 °C for 6 h and TiO_2_ (99.95%, Sigma Aldrich) at 1100 °C for 4 h. First, (001) TiO_2_ single crystal substrates (Shinkosha CO., LTD) were loaded into the PLD chamber, which was then evacuated to a base pressure of ~ 1 × 10^−6^ Torr. Then, the rotating V_2_O_5_ targets were ablated by focusing KrF excimer laser (Coherent Compex Pro 102 F, λ = 248 nm) with a fluence of 1 J/cm^2^ and repetition rate of 1 Hz. The VO_2_ growth was performed at fixed $pO_2$ = 12 mTorr and 300 °C, which was selected to induce a steep metal-insulator transition near room temperature from coherently tensile-strained VO_2_ films. After VO_2_ growth, the substrate temperature was quenched to 50 ~ 150 °C. Subsequently, TiO_2_ films were grown on VO_2_ templates under 6 mTorr ≤ $pO_2$ ≤ 24 mTorr to control the thermodynamic driving force for oxygen ionic transport across the interface at low temperature (*T*_G_ = 50–150 °C). After the growth of heterostructures, the samples were cooled down to room temperature with rate of 20 °C/min.

### Structural and electrical characterization of heterostructures

To characterize crystal-structure modulation in TiO_2_/VO_2_/TiO_2_ heterostructures with different degrees of chemical potential mismatch using $pO_2$ during TiO_2_ growth, high-resolution X-ray scattering measurements were performed using synchrotron radiation at 3A MP-XRS (*λ* ~ 0.11145 nm, energy ~ 11,125 keV at Si (111)) and at 3D XRD (*λ* ~ 0.12398 nm, energy ~ 10 keV at Si (111) beamline of Pohang Light Source-II (PLS-II, Pohang, Republic of Korea), and using an in-house HRXRD (Bruker Discover 8 X-ray diffractometer) with Cu K_α1_ radiation (*λ* = 0.15406 nm). The detailed information on in-plane and out-of-plane lattice parameters and strain states of each film in the heterostructures were obtained by using both symmetric 2θ-ω scan and asymmetric reciprocal space mapping (RSM) around the (112) reflection. The simulation of symmetric 2θ-ω scans was performed using LEPTOS software program. The surface morphology of the films was observed using an atomic force microscope (AFM, VEECO Dimension 3100).

For atomic resolution analysis of crystal structure, the samples were prepared using a dual-beam focused ion beam (FIB) system (Helios G3, FEI). HRTEM and STEM analyses (JEM-ARM200F, JEOL) were performed at 200 kV equipped with a 5^th^ order aberration corrector (ASCOR, CEOS GmbH) for forming 0.7 Å probe. The collection semi-angles were 68 to 280 mrad for HAADF, 27 to 110 mrad for LAADF and 10 to 20 mrad for ABF. The obtained raw images were band-pass filtered to reduce background noise (HREM Research Inc.).

The sheet resistance *R*_S_ was measured as a function of temperature during the heating and cooling from 250K to 340K using Hall measurement system. Measurements were carried out in van der Pauw geometry with square samples (5 mm × 5 mm) and indium Ohmic contacts (<1 mm × 1 mm) in the sample corners. The four-terminal resistances were measured using a 10-μA current.

To investigate electronic structure of TiO_2_/VO_2_ heterostructures, X-ray absorption spectroscopy (XAS) and linear dichroism (XLD) were performed using high sensitivity at the 2A MS beamline at PLS-II. The total electron yield mode with an energy resolution of ~0.1 eV was used for both measurements at a base pressure of 5 × 10^−10^ Torr in the analysis chamber by measuring the sample current (*I*_*1*_) divided by the beam current (*I*_*o*_) to remove the variation of beam intensity. The linear dichroism of V *L*_*2,3*_-edge was carried out by using horizontally-polarized or vertically-polarized X-ray beams with photon incidence angle of 22.5° at the measurement temperatures below (270 K) and above the *T*_*MI*_ (320 K) of as-grown VO_2_ films. And then, the Ti *L*_2,3_-edges XAS measurements were performed on TiO_2_/VO_2_/TiO_2_ heterostructures; photon incidence angle was 45°, and measurement temperatures were 270 K and 320 K. Due to its element-resolved characterization with several nanometer probing depth, Ti *L*_2,3_-edges spectra were obtained only from the TiO_2_ epitaxial films on top.

To evaluate the surface stoichiometry of the TiO_2_ rutile films as a result of directional oxygen transport from VO_2_ films in our TiO_2_/VO_2_/TiO_2_, XPS spectra of Ti 2*p* core level were acquired on the 4A2 SARPES and 4D PES beam line (PLS-II) in an ultra-high vacuum chamber (2 × 10^-10^ Torr). Before measurement, we carefully removed possible contaminants by using gentle Ar surface treatment. Ion sputtering was performed for 4 min and 20 min in the preparation chamber under the Ar pressure of 8 × 10^−6^ Torr at anode voltage of 500V and 2.5 kV. For collect Ti 3d spectra, we measured from 475 eV to 445 eV with 50-meV steps at 300 K. The measured spectra were deconvoluted using XPSPEAK41 software.

### First-principles calculation

First-principles density functional theory (DFT) calculations were performed using the Projector Augmented Wave (PAW) method and the generalized gradient approximation of Perdew, Burke, and Ernzerhof (PBE) for the exchange-correlation potential as implemented in Vienna Ab-initio Simulation Package (VASP) code^46^. Periodic boundary condition and Monkhorst-Pack k-point sampling with a Г-centered k-point grid of up to 8 × 8 × 8 was used for the Brillouin zone integration. An energy cutoff of 450 eV was used for the plane-wave representation of the wavefunctions and the 3s electrons of V and Ti ions were considered as valence electrons. A Hubbard U correction term was applied to the V (*U* = 3.25 eV) and Ti (*U* = 3.00 eV) to properly reproduce the strong on-site Columbic repulsion of 3d-electrons^47^. Atomic structures were relaxed until all Hellman-Feynman forces were below 0.01 eV/Å. The optimized lattice parameters are *a* = 4.67 Å and *c* = 2.52 Å for Ti metal and *a* = 3.31 Å for V metal. The optimized lattice parameters are *a* = 4.559 Å and *c* = 2.889 Å for rutile VO_2_, and *a* = 4.608 Å and *c* = 2.989 Å for rutile TiO_2_.

## Supplementary information

Supplementary Information

## Data Availability

The authors declare that the all the data supporting the finding of this study are available within this article and its Supplementary Information files, and are available from the corresponding author on reasonable request.
