# Supplementary material for: Directional ionic transport across the oxide interface enables low-temperature epitaxy of rutile TiO2
Source: Nat Commun. 2020 Mar 16;11:1401. doi: 10.1038/s41467-020-15142-x (PMC7076001; doi:10.1038/s41467-020-15142-x)
Supplement: Supplementary file 1 — Supplementary Information [file 41467_2020_15142_MOESM1_ESM.pdf]

Supplementary information for

Directional ionic transport across the oxide interface enables low-temperature epitaxy of rutile TiO_2

Yunkyu Park[1)], Hyeji Sim[1)], Minguk Jo[1)], Gi-Yeop Kim[1)], Daseob Yoon[1)], Hyeon Han[1), 3)], Younghak Kim[2)], Kyung Song[4)], Donghwa Lee[1)], Si-Young Choi[1)] and Junwoo Son[1)*]

1) Department of Materials Science and Engineering (MSE), Pohang University of Science and Technology (POSTECH), Pohang 37673, Republic of Korea
2) Pohang Accelerator Laboratory, Pohang 37673, Republic of Korea
3) Max Planck Institute of Microstructure Physics, Weinberg 2, Halle (Saale) 06120, Germany
4) Materials Modeling and Characterization Department, Korea Institute of Materials Science (KIMS), Changwon, Republic of Korea

* jwson@postech.ac.kr

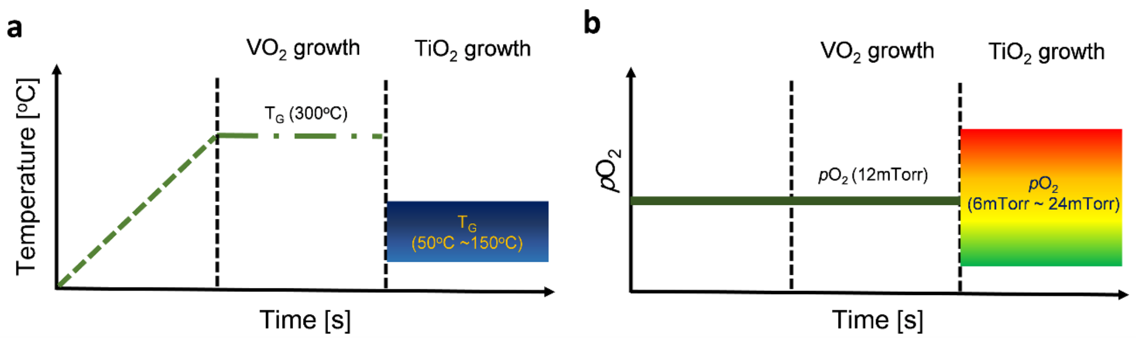

Supplementary Figure 1 | The schematic on growth process of TiO_2/VO_2 hetero-structures on TiO_2 substrates. **a.** 12nm-thick VO_2 template was grown on the (001) TiO_2 substrate with 12mTorr oxygen pressure at 300ºC. After preparation of VO_2 template, rutile TiO_2 thin films were grown by **b.** changing oxygen partial pressure from 6mTorr to 24mTorr at low temperature (T_G = 50 ~ 150 °C). By adjusting P_{O_2} during TiO_2 growth, we successfully grew TiO_2/VO_2 heterostructures with modulating oxygen chemical potential between TiO_2 and VO_2.

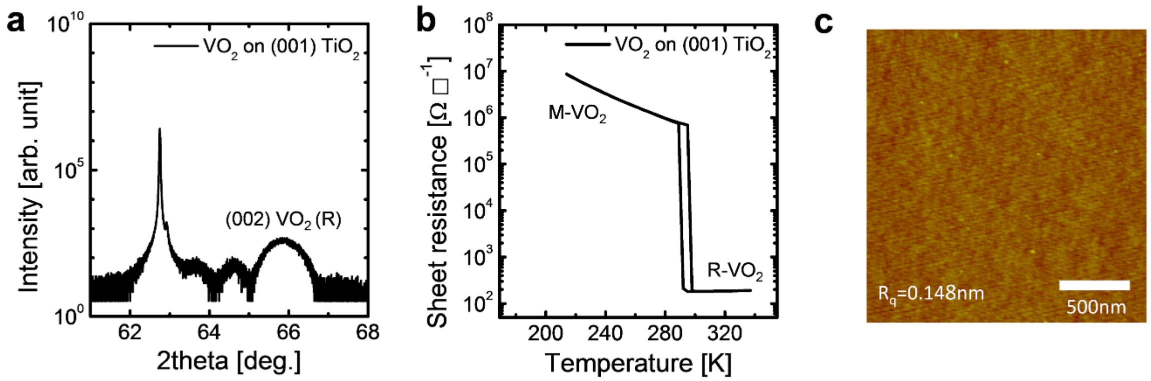

Supplementary Figure 2 | **a.** Symmetric X-ray scan of the optimized as-grown VO_2 templates grown on (001) TiO_2 substrate. **b.** Steep transition of temperature-dependent sheet resistance in VO_2 templates on (001) TiO_2 substrate. **c.** AFM image of flat VO_2 surface with low $R_q \sim 0.148$nm. These results indicate that initial VO_2 layers exhibit good stoichiometry and high crystal quality.

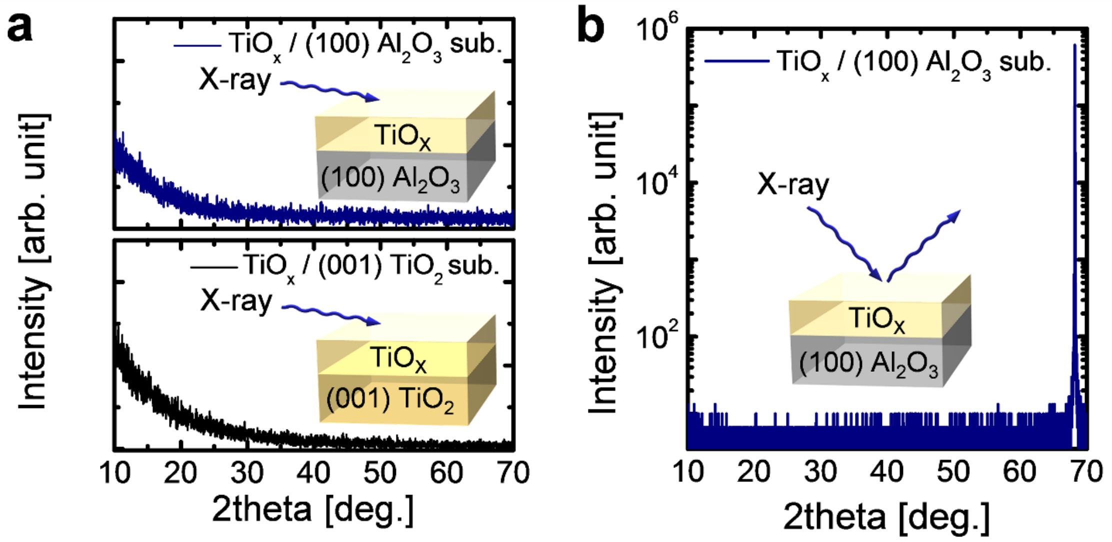

Supplementary Figure 3 | **a.** Grazing-incidence x-ray diffraction to characterize crystalline phase in TiO_x film. No obvious peak was observed in both TiO_x / (100) Al_2O_3 substrate and TiO_x / (001) TiO_2 substrate. **b.** wide range scan (10° ~ 70°) of symmetric 2θ-ω scans of TiO_x films grown at 150 °C on (100) Al_2O_3 substrate. Only peak related to the *m*-Al_2O_3 substrate was detected, which strongly support the formation of amorphous TiO_2 layer without VO_2 template grown at 150 °C.

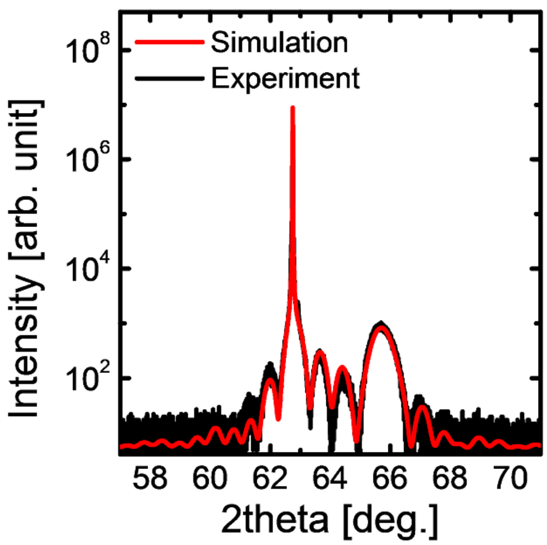

Supplementary Figure 4 | Fitting of symmetric 2θ-ω scan of high-resolution x-ray diffraction measurement from TiO_2/VO_2 hetero-structure for the data shown in Fig. 1d. The fitting was performed with the Bruker LEPTOS software program.

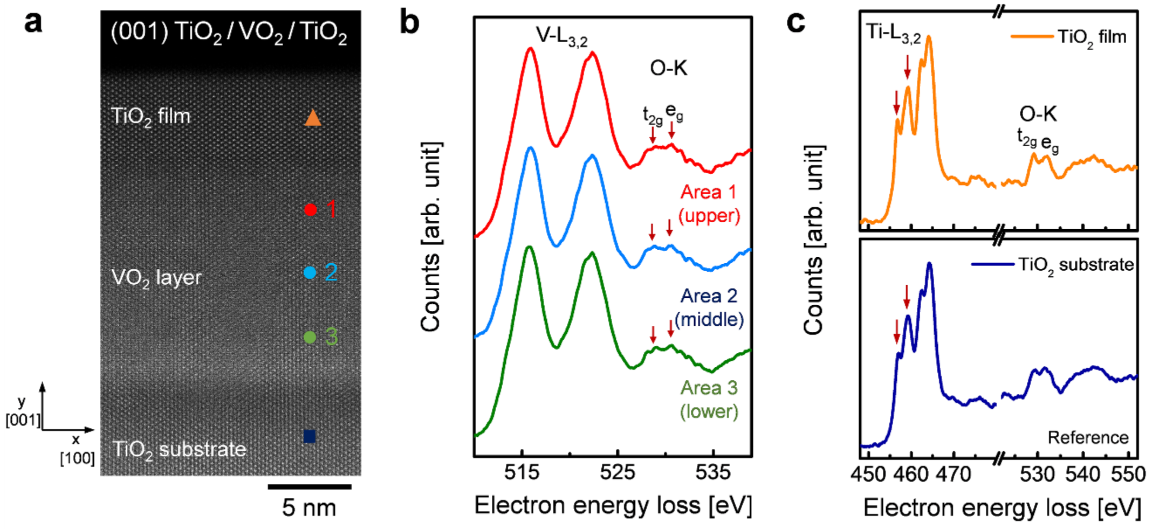

Supplementary Figure 5 | a. HAADF image and **b.** V-$L_{2,3}$ edge, O-*K* edge spectra from VO_2 layer and **c.** Ti-$L_{2,3}$ edge, O-*K* edge from TiO_2 layers through the cross-sectional positions in our $(001)_R$-oriented TiO_2/VO_2 hetero-layers grown at 150 °C by using EELS (Electron Energy Loss Spectroscopy). Obviously, t_{2g} peaks of O-*K* edges from the top (**1** in **a**), middle (**2** in **a**), and bottom areas (**3** in **a**) of VO_2 layers are all suppressed. This suppressed t_{2g} peak of O-*K* edge is due to the oxygen vacancy induced valence reduction of V ions, and therefore the oxygen vacancies and lower valence state of V ions are clearly formed in the entire VO_2 layers. **c.** The t_{2g} peaks of Ti-*L* edge from TiO_2 layers is exactly same as those from TiO_2 substrates (reference).

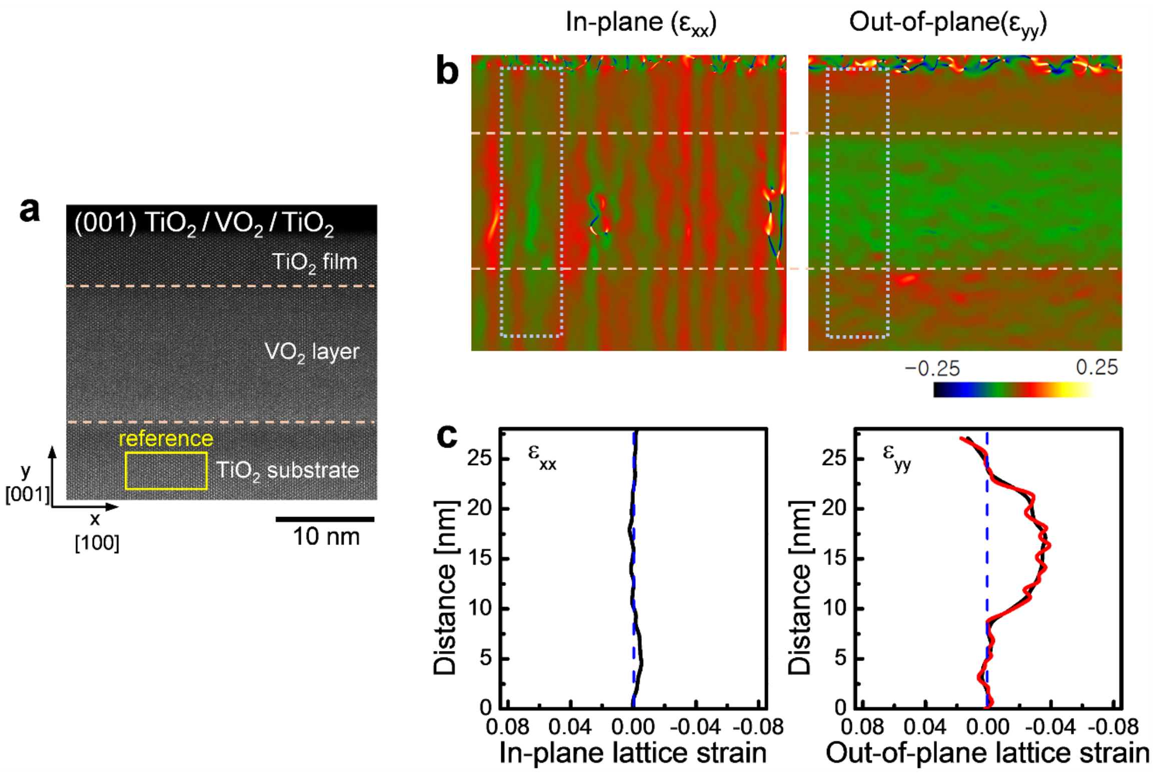

Supplementary Figure 6 | a. HAADF-STEM image of $(001)_R$-oriented TiO_2/VO_2 heterostructure grown at 150 °C on (001) TiO_2 substrates. **b.** In-plane (ε_{xx}) and out-of-plane geometrical phase analysis (GPA) of HAADF-STEM images. **c.** line strain profile of in-plane lattice and out-of-plane lattice in GPA images. Almost negligible variation of in-plane lattice parameter was detected across the entire $TiO_2/VO_2/TiO_2$ hetero-structures, which confirms coherent interfaces through the hetero-structures. On the other hand, our-of-plane lattice parameters of TiO_2 layers are almost identical to those of reference (TiO_2 substrates). Thus, TiO_2 films in heterostructures maintain bulk lattice parameters, which exhibit no significantly different contribution of interfacial strain energy to ΔG^* during the TiO_2 growth on VO_2 templates, compared to TiO_2/TiO_2 heterostructures.

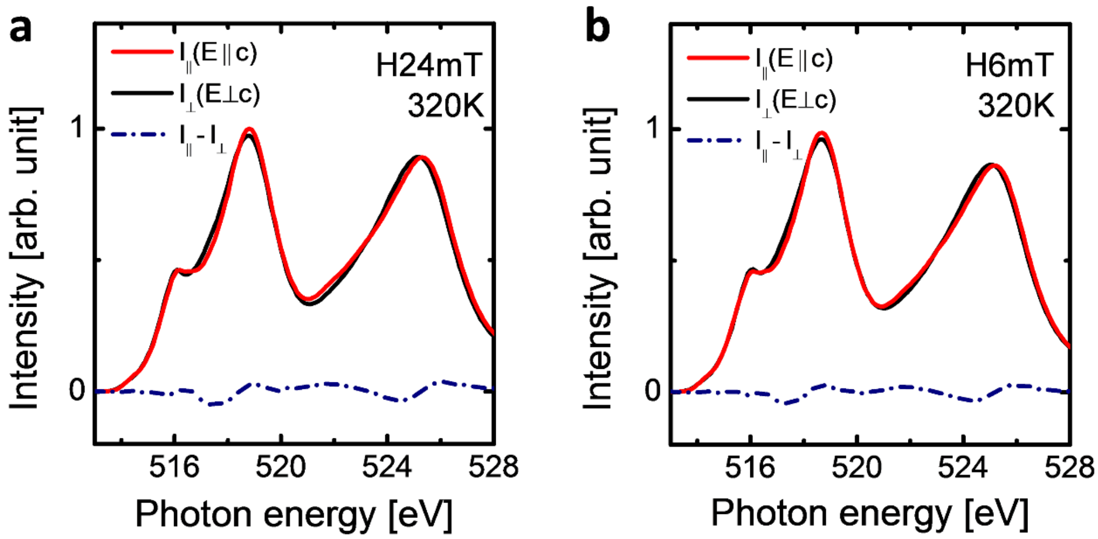

Supplementary Figure 7 | Polarization-dependent XAS spectra of V $L_{2,3}$-edge at 320 K. **a.** H24mT and **b.** H6mT identically show isotropic orbital occupation at 320 K. XAS spectra collected at 320 K ($T > T_{MI}$) were similar in both samples regardless of the polarization direction of the X-ray; this result was expected because of the isotropic orbital filling in the metallic states of VO_2 at 320 K due to absence of V-V dimerization.

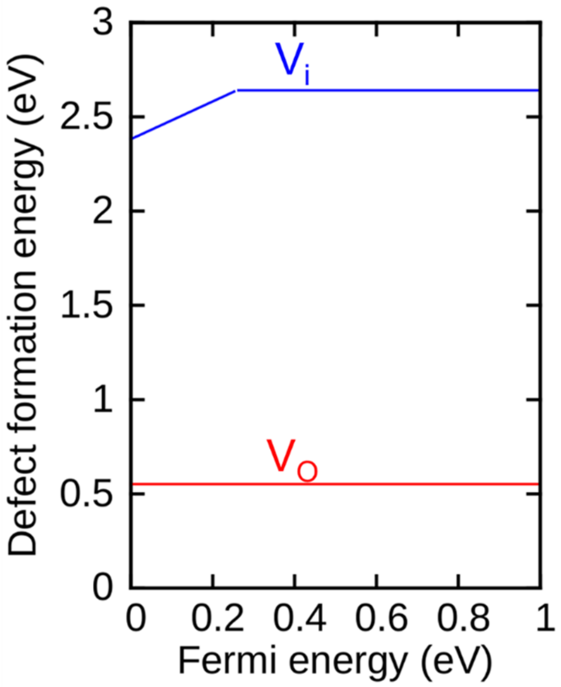

Supplementary Figure 8 | Comparison between formation energy of oxygen vacancy (red) and vanadium interstitial (blue) in VO_2. The formation energy of V interstitial (~ 2.5 eV) is much higher than that of O vacancy (~ 0.5 eV). Thus, we believe that the formation of O vacancies is much preferable in VO_2.

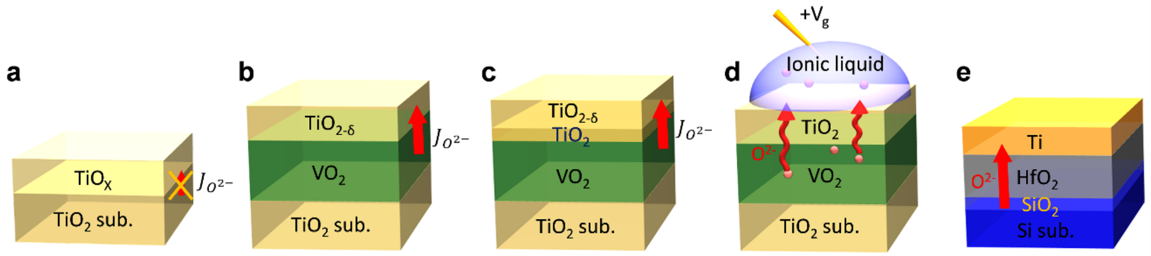

Supplementary Figure 9 | Schematic figures of **a.** homo-growth of TiO_x on TiO_2 substrate with negligible oxygen ionic transport, **b.** hetero-growth of $TiO_{2-\delta}$ on VO_2/TiO_2 substrate and **c.** heteroepitaxial growth of $TiO_{2-\delta}$ after the growth of few TiO_2 monolayers on VO_2/TiO_2 substrate. Although few TiO_2 monolayers are inserted, the driving force for oxygen flux is still formed across the interface between $TiO_{2-\delta}$ and VO_2. The combination of facile thermodynamics and kinetics, "remote" transport of oxygen ion through intervening TiO_2 layer is highly probable during the growth of $TiO_{2-\delta}$; Oxygen vacancies can be induced remotely and persistently even after initial formation of TiO_2 layers, as long as oxygen diffusion is allowed across the pre-formed TiO_2 layers. The example of "remote" oxygen transport **d.** through intervening TiO_2 insulating layer induced by electrochemical potential across the ionic liquid (*Nano Lett.* 16, 5475-5481 (2016)) and **e.** from SiO_2 interlayer to TiO_x overlayer through the intervening HfO_2 as reported previously (*J. Appl. Phys*., 96, 3467-3472 (2004)).

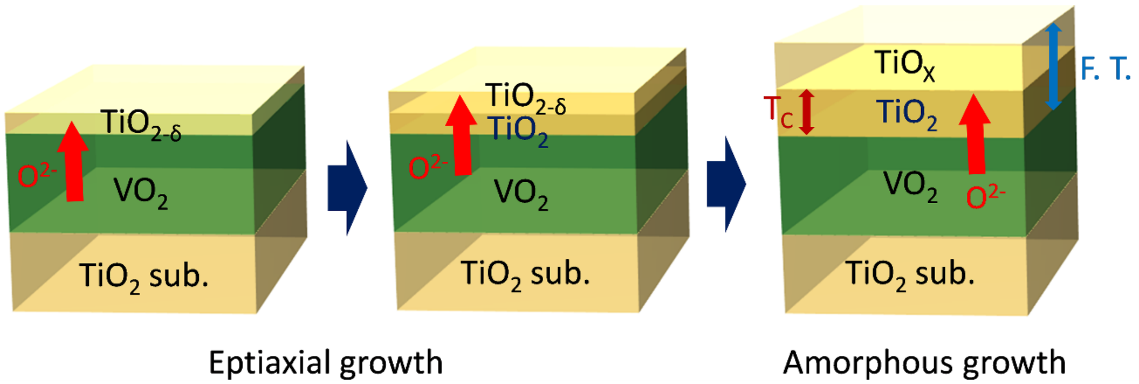

Supplementary Figure 10 | The existence of a "critical" thickness (T_c) for epitaxial growth based on "internal" oxygen transport across the TiO_2/VO_2 interface. Since the oxygens should be supplied from the TiO_2/VO_2 interfaces through the intervening TiO_2 layers, the thickness of epitaxial TiO_2 (E.T.) will be limited up to the "critical" thickness (T_c) by oxygen diffusion through the intervening TiO_2 layer, even if the growth time (g. t.) increases to increase film thickness (F. T.).

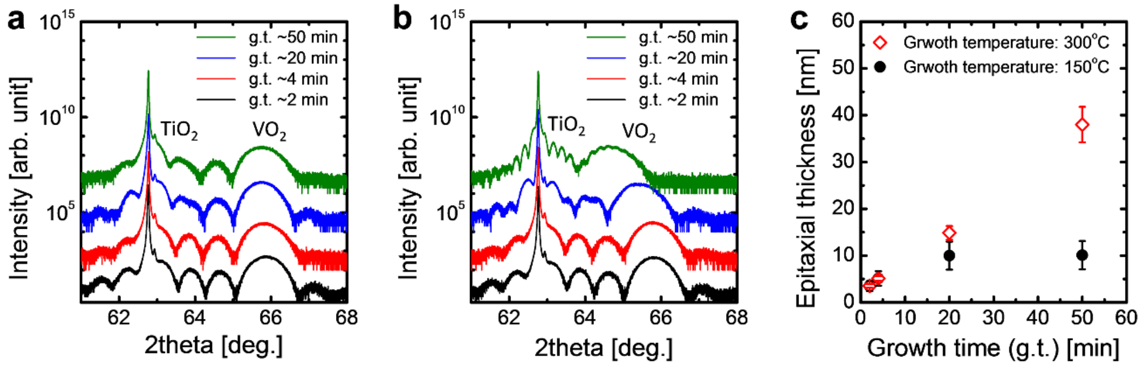

Supplementary Figure 11 | Symmetric 2θ-ω XRD scans of all TiO_2 films with various growth time (i.e., film thickness) grown on VO_2/TiO_2 substrates at two different temperature (**a.** 150 °C and **b.** 300 °C). **c.** After the simulation of X-ray diffraction peak and Kiessig fringes by LEPTOS, the "epitaxial thickness" was extracted and plotted as a function of the growth time of TiO_2 layers. The feature of Kiessig fringes (i.e., the signature of epitaxial growth) was saturated even with longer growth time (at thicker "film" thickness) grown at 150 °C; the formation of epitaxial TiO_2 films are limited by oxygen diffusion through the intervening TiO_2 layer above the "critical" thickness (~ 10 nm for T_G = 150 °C).

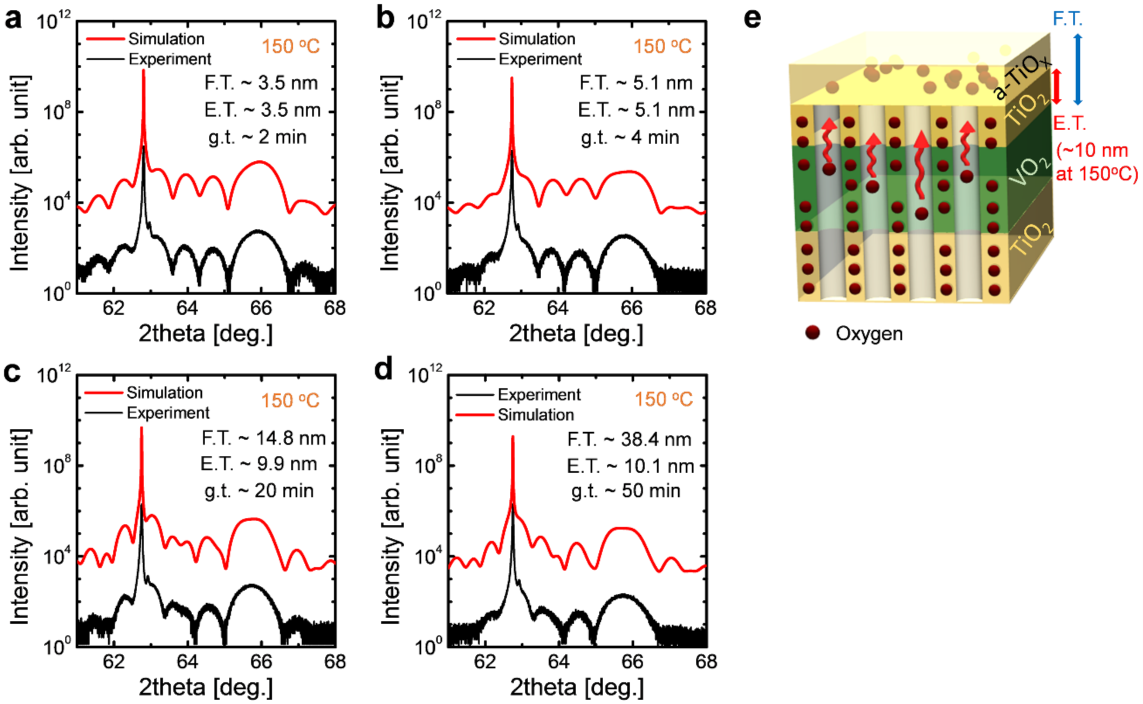

Supplementary Figure 12 | The simulated and experimental results of the X-ray diffraction peak and Kiessig fringes by LEPTOS to estimate epitaxial thickness of TiO_2 films with different growth time (**a.** 2, **b.** 4, **c.** 20, **d.** 50 min) on (12 nm) VO_2/TiO_2 in all heterostructures in Supplementary Figs. 11a. e. The epitaxial thickness for TiO_2 films was eventually saturated by critical thickness at (~ 10 nm) even after 50 min growth at 150 °C, which shows the existence of "critical" thickness of epitaxial growth and the convincing evidence of our proposed growth mechanism.

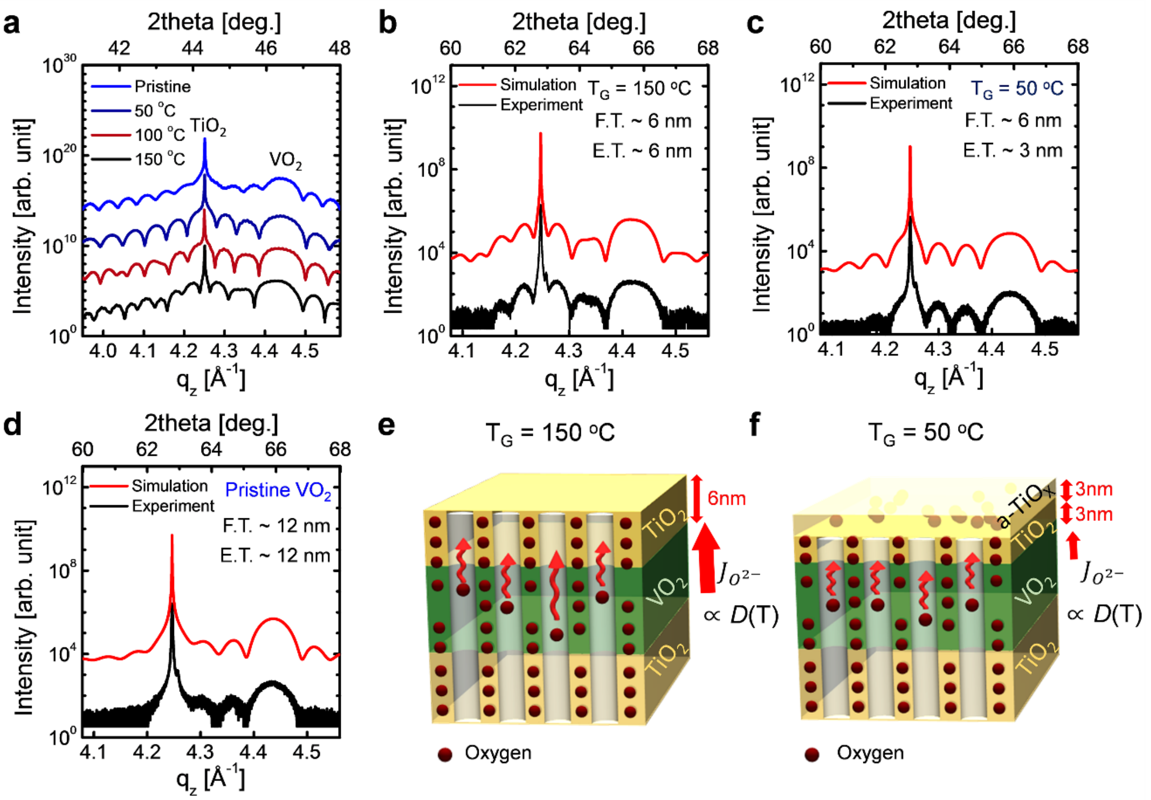

Supplementary Figure 13 | a. Symmetric 2θ-ω XRD scans (using synchrotron x-ray scattering (λ ~ 0.11145nm, energy ~ 11.125keV) of all TiO_2 films with various growth temperature (T_G = 50 °C, 100 °C, 150 °C) grown on (12 nm) VO_2/TiO_2 substrates with same growth time. The comparison of simulations and experiments of symmetric 2θ-ω scans TiO_2/VO_2 hetero-structures with different T_G (**b.** T_G = 150°C, **c.** T_G = 50°C) and **d.** pristine VO_2 films on (001) TiO_2 substrate. The epitaxial thickness of TiO_2 film are reduced from ~ 6 nm (150 °C) to ~ 3 nm (50 °C) even at the same growth time. **e, f.** The thickness of "epitaxial" growth was kinetically limited by the growth temperature of TiO_2 films, which will determine the temperature-dependent oxygen ionic flux ($J_{O^{2-}} \propto$ D(T)) by ionic diffusion from VO_2 to TiO_2 films. The "critical" thickness for epitaxial growth was further reduced down to ~ 3 nm for the growth at 50 °C.

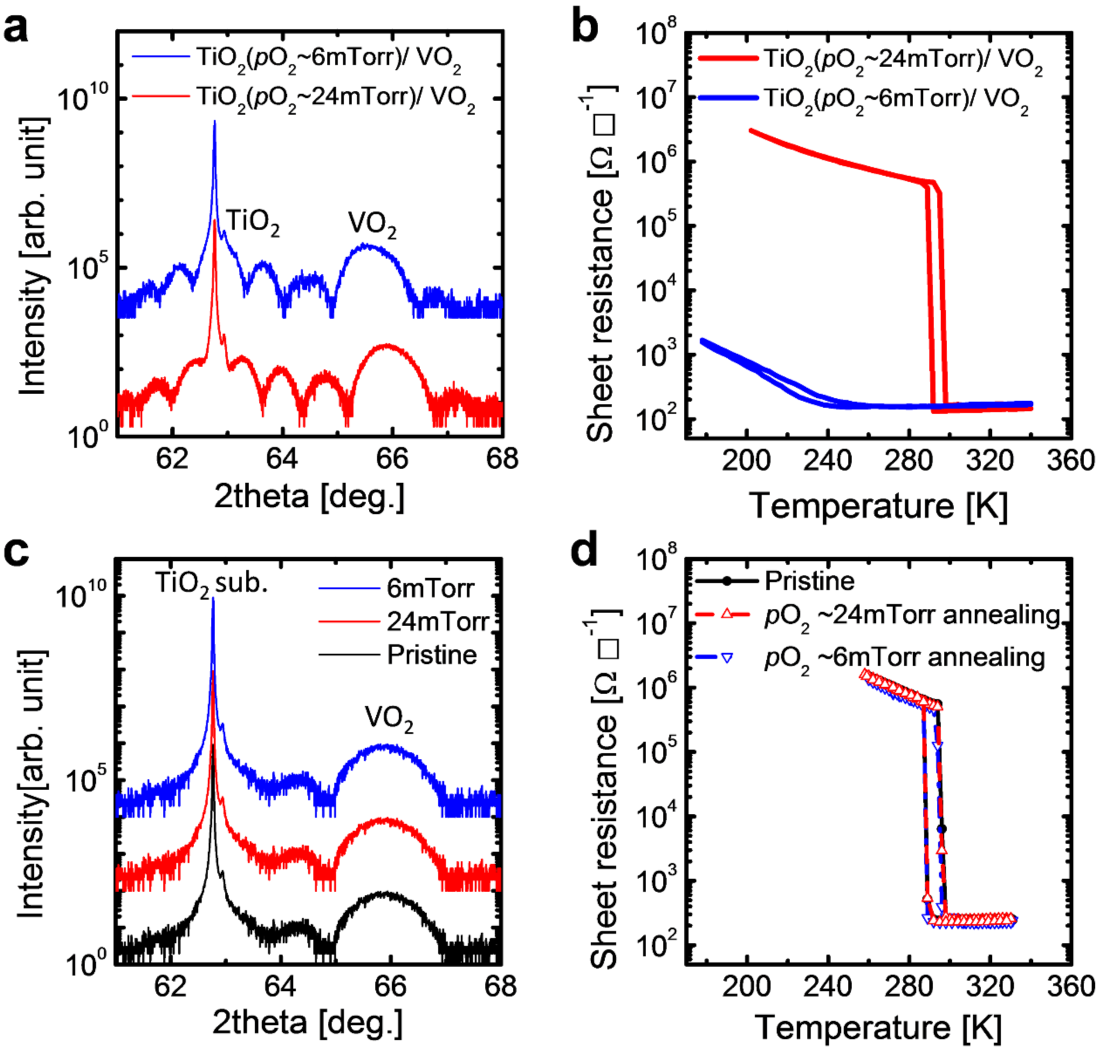

Supplementary Figure 14 | Structural and electrical modulation of **a, b.** VO_2 templates by adjusting oxygen partial pressure (i.e., pO_2 = 6 mTorr and 24 mTorr at 150°C) during TiO_2 growth and **c, d.** post-annealed VO_2 templates under the TiO_2 growth condition (i.e., pO_2 = 6 mTorr and 24 mTorr at 150°C) without growing TiO_2 layer. While the expanded out-of-plane lattice parameter (**a**) and suppressed MI transition (**b**) of VO_2 template was observed by reducing pO_2 during TiO_2 growth, simple post-annealing of VO_2 films show the negligible change on structural (**c**) and electrical (**d**) properties. Thus, oxygen deficiency in VO_2 layer cannot be generated without TiO_2 layer growth on top, and thus directional oxygen ionic transport indeed occurs across the TiO_2/VO_2 interfaces by forming oxygen deficiency in VO_2 layer, only when TiO_2 layers are grown on VO_2 templates.

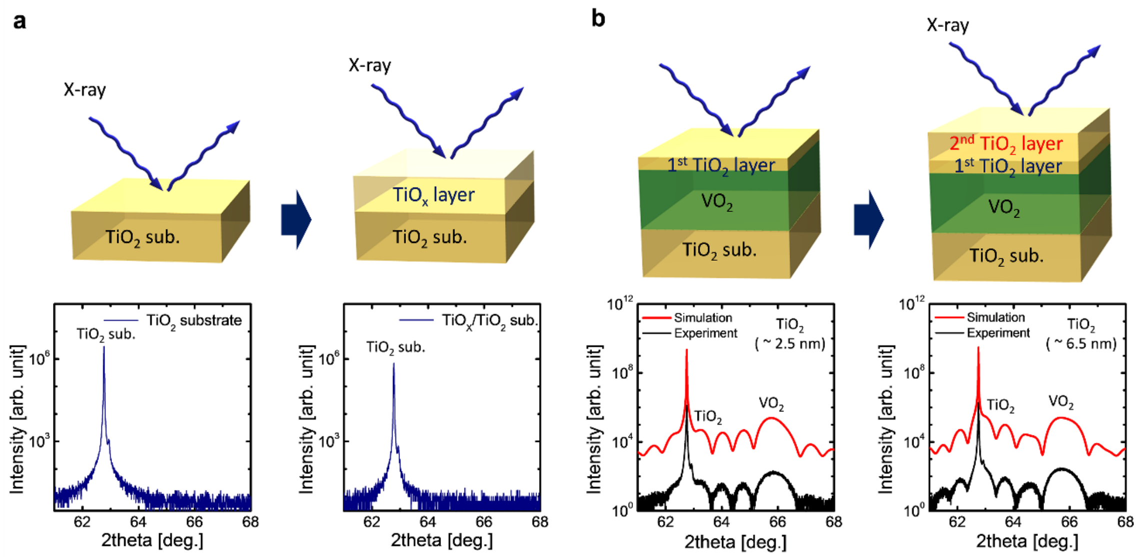

Supplementary Figure 15 | Additional experiments to exclude the effect of surface free energy by making identical TiO_2 surfaces for the TiO_2 film growth. **a.** TiO_2 film growth on TiO_2 substrates for homo-structures. **b.** TiO_2 film growth on (~2.5 nm) $TiO_2/VO_2/TiO_2$ substrates. Except the interface between TiO_2/VO_2 in **b**, two samples exhibit the identical TiO_2 surface exposure during the TiO_2 growth. Unlike the amorphous formation of TiO_x films on TiO_2 substrates (no TiO_2 epitaxial film XRD peak around substrate in **a**), epitaxial rutile TiO_2 growth thicker than initial a few layer TiO_2 (~ 6.5 nm) was still observed on (~ 2.5 nm) $TiO_2/VO_2/TiO_2$ hetero-structures (XRD in **b**). Thus, completely different crystal growth of TiO_2 on two types of TiO_2 surfaces (i.e., on TiO_2 substrates and on (~ 2.5 nm) $TiO_2/VO_2/TiO_2$ hetero-structures) clearly exclude the influence of surface free energy on the ΔG^* between homo-structure and hetero-structure during the TiO_2 growth.

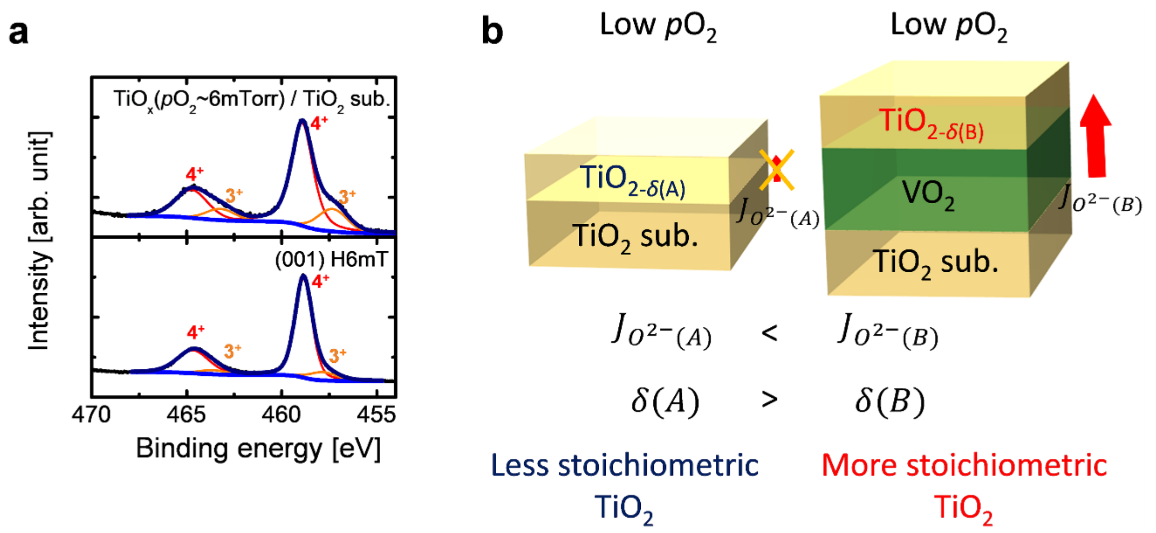

Supplementary Figure 16 | a. XPS Ti 2*p* core level spectra of TiO_2/TiO_2 homo-structure and TiO_2/VO_2 hetero-structure (denoted as (001) H6mT) after the TiO_2 film growth under P_{O_2} ~ 6 mTorr at 150 °C. **b.** The increased "effective" P_{O_2} by the enhanced $J_{O^{2-}}$ across the interface magnifies driving force for the formation of rutile TiO_2 with stoichiometry with higher Ti^{4+} contribution in heterostructure (characterized by XPS (**a**)).
